# Nucleotide Binding Switches the Information Flow in Ras GTPases

**DOI:** 10.1371/journal.pcbi.1001098

**Published:** 2011-03-03

**Authors:** Francesco Raimondi, Guillem Portella, Modesto Orozco, Francesca Fanelli

**Affiliations:** 1 Department of Chemistry, University of Modena and Reggio Emilia, Modena, Italy; 2 Dulbecco Telethon Institute (DTI), University of Modena and Reggio Emilia, Modena, Italy; 3 Molecular Modeling and Bioinformatics Unit, IRB-BSC Joint Research Program in Computational Biology, Institute for Research in Biomedicine, and Barcelona Supercomputing Center, Barcelona, Spain; 4 National Institute of Bioinformatics, Parc Científic de Barcelona, Barcelona, Spain; Fox Chase Cancer Center, United States of America

## Abstract

The Ras superfamily comprises many guanine nucleotide-binding proteins (G proteins) that are essential to intracellular signal transduction. The guanine nucleotide-dependent intrinsic flexibility patterns of five G proteins were investigated in atomic detail through Molecular Dynamics simulations of the GDP- and GTP-bound states (S^GDP^ and S^GTP^, respectively). For all the considered systems, the intrinsic flexibility of S^GDP^ was higher than that of S^GTP^, suggesting that Guanine Exchange Factor (GEF) recognition and nucleotide switch require higher amplitude motions than effector recognition or GTP hydrolysis. Functional mode, dynamic domain, and interaction energy correlation analyses highlighted significant differences in the dynamics of small G proteins and Gα proteins, especially in the inactive state. Indeed, S^GDP^ of Gα_t_, is characterized by a more extensive energy coupling between nucleotide binding site and distal regions involved in GEF recognition compared to small G proteins, which attenuates in the active state. Moreover, mechanically distinct domains implicated in nucleotide switch could be detected in the presence of GDP but not in the presence of GTP. Finally, in small G proteins, functional modes are more detectable in the inactive state than in the active one and involve changes in solvent exposure of two highly conserved amino acids in switches I and II involved in GEF recognition. The average solvent exposure of these amino acids correlates in turn with the rate of GDP release, suggesting for them either direct or indirect roles in the process of nucleotide switch. Collectively, nucleotide binding changes the information flow through the conserved Ras-like domain, where GDP enhances the flexibility of mechanically distinct portions involved in nucleotide switch, and favors long distance allosteric communication (in Gα proteins), compared to GTP.

## Author Summary

The Ras superfamily comprises many guanine nucleotide-binding proteins (G proteins) that are essential to intracellular signal transduction. These proteins act biologically as molecular switches cycling between ON and OFF states, thereby controlling a variety of processes ranging from cell growth and differentiation to vesicular and nuclear transport. In spite of the extremely high biological and medical relevance of the Ras GTPase superfamily, a comprehensive structural/dynamic view of the trans-family and family-specific functioning mechanisms is still lacking. In this study, we gained insights into the functional dynamics of Ras GTPases by deciphering the dynamic information encrypted in the topology of these proteins depending on the nucleotide-bound state, i.e. GDP- or GTP-bound (S^GDP^ and S^GTP^, respectively). Collectively, nucleotide binding changes the information flow through the conserved Ras-like domain, where GDP enhances the flexibility of mechanically distinct portions involved in nucleotide switch, and favors long distance allosteric communication (in Gα proteins), compared to GTP. Functional dynamics is instrumental in GDP switch, which for the members of the Gα family, different from small G proteins, requires allosteric communication between nucleotide and Guanine Exchange Factor binding sites.

## Introduction

The Ras superfamily comprises many guanine nucleotide-binding proteins (G proteins) that are essential to intracellular signal transduction [1], [2]. These proteins act biologically as molecular switches cycling between ON and OFF states, thereby controlling a variety of processes ranging from cell growth and differentiation to vesicular and nuclear transport [1]. The switch-on process requires the release of the bound Guanosine Di-Phosphate (GDP) and the subsequent binding of the Guanosine Tri-Phosphate (GTP), an intrinsically slow process catalyzed by Guanine nucleotide Exchange Factors (GEFs) [1]. In the GTP-bound active state (S^GTP^), the G proteins display high affinity for binding downstream effectors, interactions through which they exert their specific biologic functions. The switch-off process involves the hydrolysis of GTP to GDP, reaction that is accelerated by Guanine nucleotide Activating Proteins (GAPs) and leads to release of effector proteins (due to reduced affinity) and attenuation of downstream signaling [1]. Peculiar to the members of the Gα family is the fact that in the inactive GDP-bound state (S^GDP^) they form membrane-associated αβγ heterotrimers, with GDP bound to the α-subunit [3].

**Figure 1:**
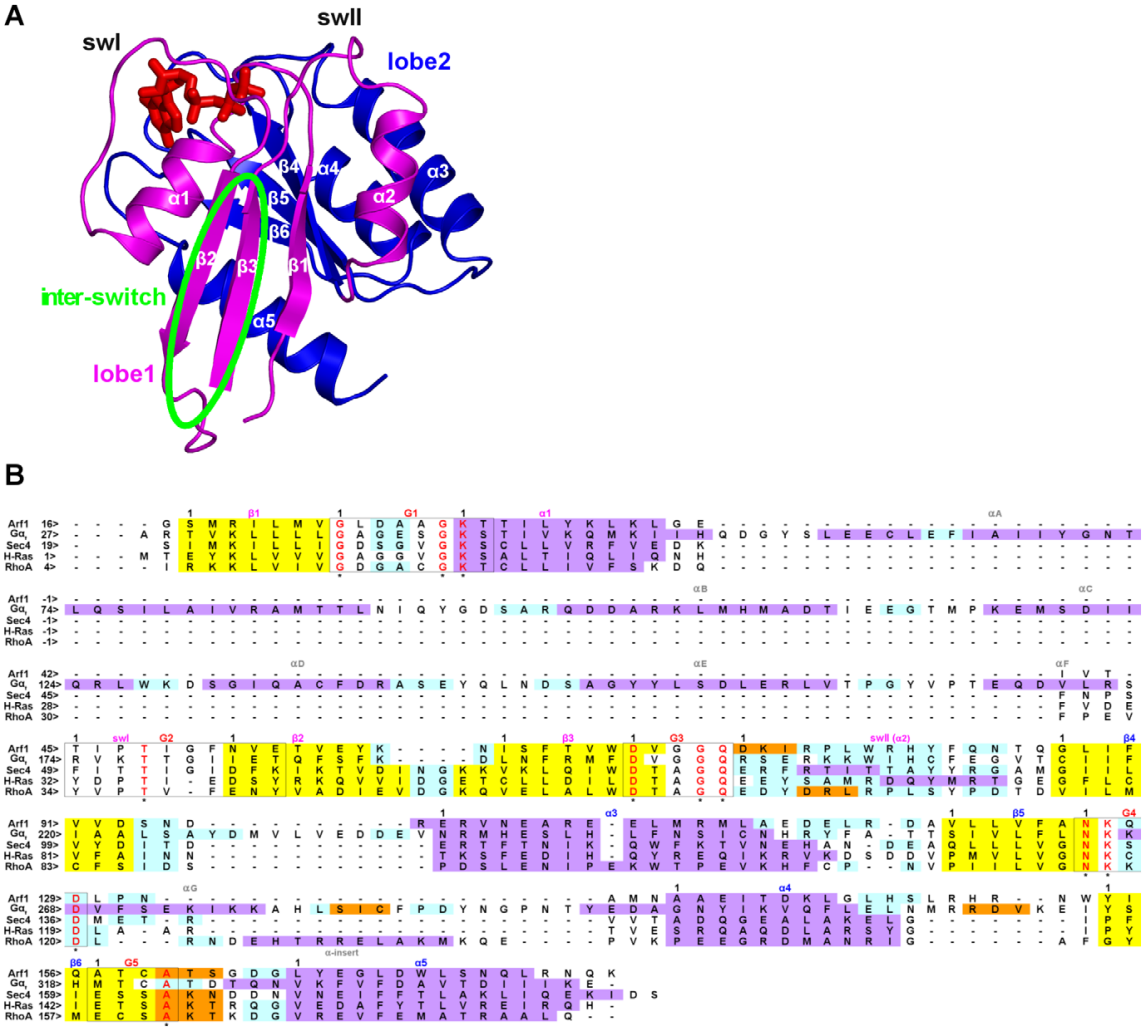
Structure and sequence features of the five GTPases. **A**: cartoons of the H-Ras structure (PDB code: 5P21) in its GTP-bound state are shown. The Ras superfamily GTPases share a common domain, the Ras-like domain. The latter, according to CATH [34], is characterized by a Rossmann fold with a 3-layer(αβα) sandwich architecture, where helices 1 and 5 (α1 and α5; the secondary structure elements in the Ras-like domain are labeled according to the Noel's nomenclature [35]) lay on one side, whereas α2, α3, and α4 lay on the other side of the central five-stranded parallel β-sheet (i.e. comprising the β1 and β3-β6 strands, Figures 1 and 2). The helices α1 and α3 lay on the opposite side of the sheet due to the inversion in the order of the preceding strands, β1 and β3, respectively, which are adjacent to each other. The β1/α1 loop, i.e. phosphate binding loop (P loop), and the region comprising α2 as well as the preceding and following loops (i.e. switch II (swII)) participate in the binding of the nucleotide phosphates (Figures 1 and 2). The architecture of this superfamily is such that β1 is also adjacent to β4. The β1/β4 interface divides the Ras-like domain into two lobes: i) lobe 1 (i.e. the N-terminal half of the domain, magenta) includes the β1-β3 strands, the P-loop and the two switches, and ii) lobe 2 (blue), which includes the β4-β6 strands and the α3-α5 helices. Another structural feature of the conserved Ras domain is that β2 forms a β-hairpin with β3, the loop that connects the two antiparallel strands being directed towards the opposite side of the nucleotide binding cleft (Figures 1 and 2) [3]. The β2/β3 hairpin is also called “inter-switch” (i.e. delimited by a green oval) because the loops that enter β2 and exit from β3 constitute, respectively, the swI and swII regions. The loops connected to the C-term of β1 and the N-term of β2, P loop and swI, respectively, define most of the nucleotide binding site. The members of the Gα family hold an extra-Ras α-helical domain constituted by a long central helix surrounded by five shorter helices. The interface between α-helical and Ras-like domain constitutes the nucleotide binding cleft. Incidentally, among the small G proteins RhoA has a structural peculiarity consisting of a ten amino acid α-helical insertion (α-insert) on the β5/α4 loop like the αG segment shared by the members of the Gα family. B): the multiple sequence alignment derived from the multiple structure alignment of representatives of the S^GTP^ state of Arf1 (PDB code: 1O3Y), Gα_t_ (PDB code: 1TND), Sec4 (PDB code: 1G17), H-Ras (PDB code: 5Q21), and RhoA (PDB code: 1KMQ) is shown (i.e. achieved by the Multiprot-Staccato software) [36]. Helices, strands, and loops are, respectively, violet, yellow, and cyan. Ultra-conserved sequences involved in nucleotide binding (G boxes) are delimited by black boxes. Black numbers on the left side of the alignment refer to the sequential numbering, whereas black numbers above the sequences indicate the beginning of a secondary structure/G box motif. The fully conserved residues in such boxes are red and marked by an asterisk. In order to facilitate trans-family comparisons of the MD simulation outputs, an arbitrary numbering was set characterized by the label of the secondary structure segment followed by the amino acid position in that segment. In those cases where the G-boxes overlap with the secondary structure segment, positions refer to the G-boxes.

**Figure 2:**
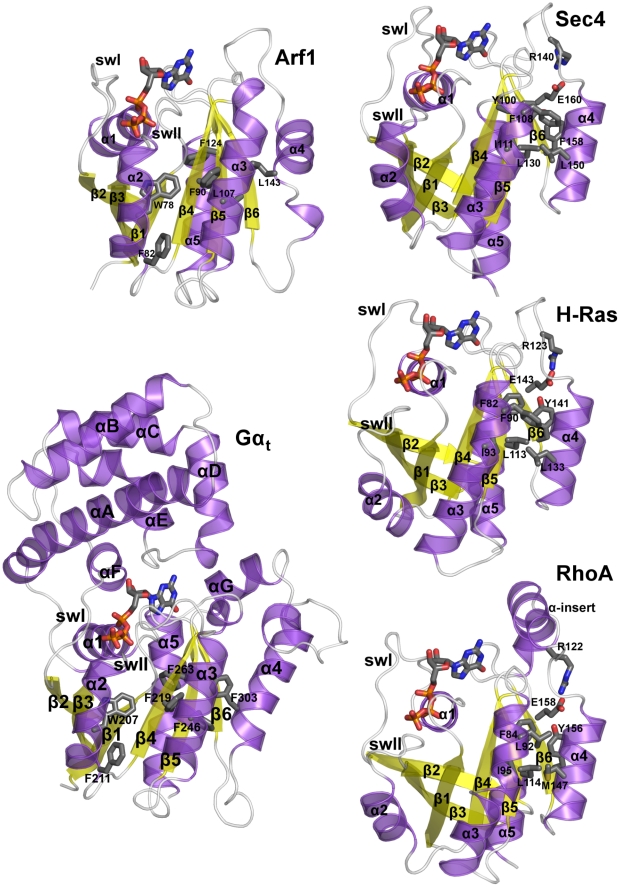
Structural features of the five GTPases. Cartoons of the S^GTP^ state of Arf1 (PDB code: 1O3Y), Gα_t_ (PDB code: 1TND), Sec4 (PDB code: 1G17), H-Ras (PDB code: 5Q21), and RhoA (PDB code: 1KMQ) are shown. The structures are colored according to secondary structure. The nucleotide is represented by sticks colored by atom type. Selected side chains of amino acids conserved in groups of G protein families are shown by sticks. Structural analysis, indeed, reveals clusters of conserved amino acids shared by selected family members. In particular, Sec4, H-Ras, and RhoA share a cluster of conserved aromatic/hydrophobic amino acids at positions β4∶6, α3∶4, α4∶7, and α4∶11 as well as a glutamate in position G5∶1, which is engaged in a salt bridge with an arginine on the β5/α4 loop that holds the same conservation pattern of the glutamate. In contrast, Arf1 and Gα_t_ share a cluster of hydrophobic/aromatic amino acids at positions β4∶4, α3∶13, and α4∶8 (Figure 2). Another feature that distinguishes Arf1 and Gα_t_ from the other three G proteins is the α4/β6 loop that is significantly longer in the former.

Nucleotide switch and hydrolysis in Ras GTPases are played by the conserved core, Ras-like domain (see Figure 1 legend for structure description). Selected members of the superfamily, such as the members of the Gα family, hold an extra-Ras α-helical domain (Figures 1 and 2) constituted by a long central helix surrounded by five shorter helices. This feature makes Gα proteins significantly larger than all the other members of the Ras superfamily, which are, hence, indicated as “small G proteins”.

The central role of Ras GTPases in cell function is testified by the proved involvement of selected members like Ras and Rho in many aspects of cancer development and tumor progression, which makes these proteins very interesting targets in cancer therapy [2]. This is why oncogenic Ras mutants have been the target of computational experiments aimed at unraveling the dynamic information encoded into the structure [4], [5].

In a recent study, we combined Elastic Network Model (ENM) coarse grained simulations with Principal Component Analysis (PCA) on the experimental structures of representatives Ras GTPases to decipher the physical and evolutionary deformability patterns that enable switching between active and inactive states [6], [7]. The analysis highlighted functional separation of the conserved core into two lobes, as previously suggested by others [5], [8]. The deformation modes involved in the switching function are conserved along evolution and are localized in lobe 1 portions close to the nucleotide (Figure 1). These modes lead to functional specialization when associated with evolution-driven deformations of protein portions essentially located in lobe 2, distal from the nucleotide, and involved in specific interactions with membrane, GEFs, or effectors [6]. Additional evidence that the Ras superfamily members share a set of switching dynamics was inferred by the identification of conserved hinge points throughout all subfamilies, which remark the bi-lobate dynamics of the conserved core [6].

In this study, we analyze in atomistic detail the intrinsic flexibility patterns of G proteins through Molecular Dynamics (MD) simulations of selected members of the five major families of Ras GTPases (i.e. Arf1, Gα_t_ (also called transducin), Sec4, H-Ras, and RhoA). Functional dynamics of these molecular switches was investigated through the analyses of essential motions, functional modes, interaction energy correlations and dynamic domains in relation to the functional states, i.e. S^GDP^ or S^GTP^. Our simulations confirm the complexity of the deformability patterns of Ras GTPases and the careful tuning that evolution has made on it. In G proteins, functional dynamics is suggested to be instrumental in GDP switch, which, for the members of the Gα family, different from the small G proteins, requires allosteric communication between nucleotide and GEF binding sites [3], [9], [10].

## Results

### Analysis of the intrinsic flexibility of Ras GTPases

**Figure 3:**
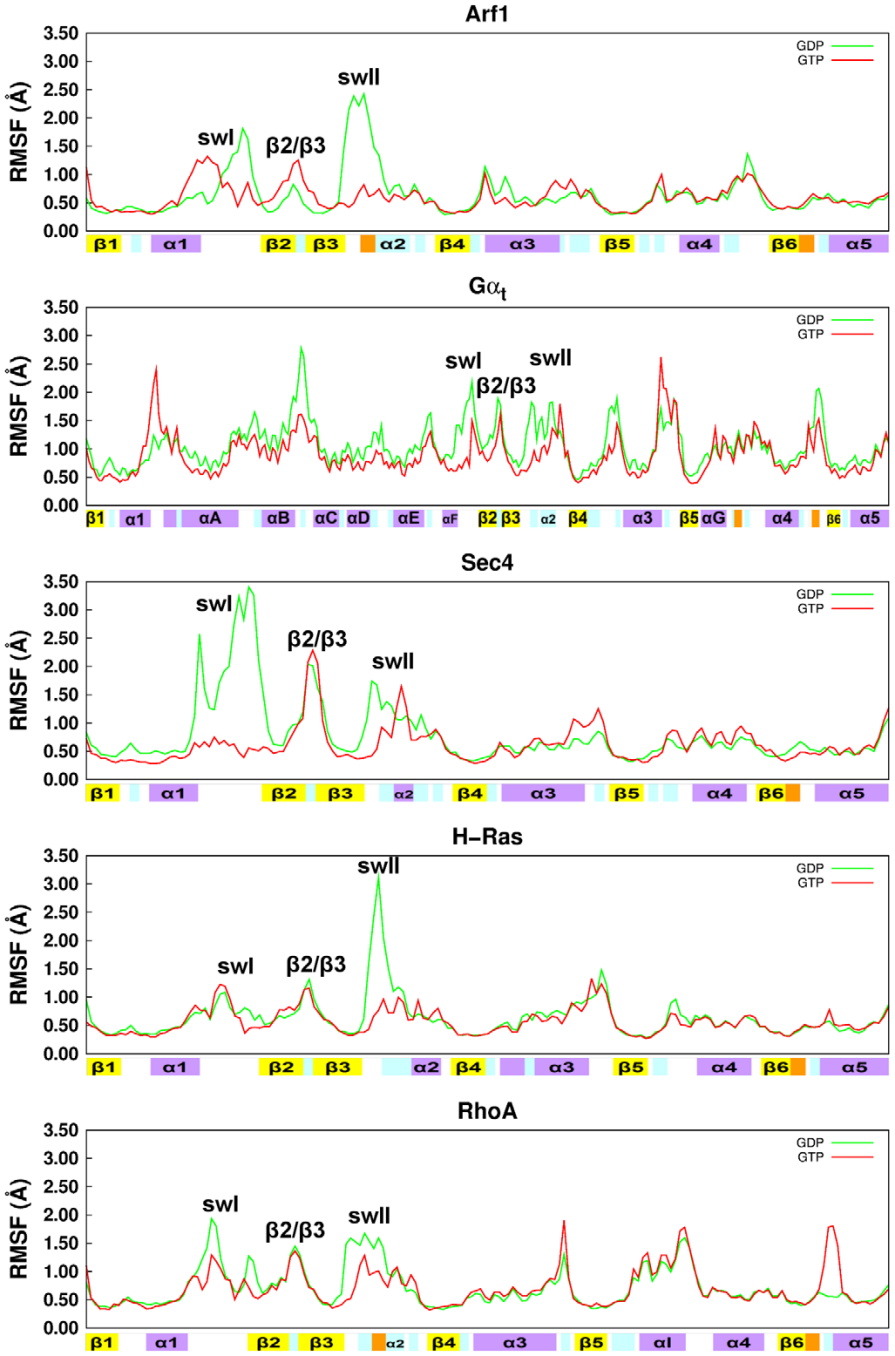
RMSF profile from MD trajectories of the S^GDP^ and S^GTP^ forms of the five Ras GTPases. Green and red lines refer to the S^GDP^ and S^GTP^ forms, respectively, of Arf1, Gα_t_, Sec4, H-Ras, and RhoA. RMSF profiles refer to the 40000 frames constituting 40 ns trajectories. The secondary structure elements are shown on the abscissa, following nomenclature and color code described in Figure 1.

Remarkable functional-state dependent features shared by the five homologous GTPases reside in the Cα-Root Mean Square Fluctuation (Cα-RMSF) profiles, showing higher flexibility for the inactive forms compared to the active ones, with swI and swII being the lobe 1 portions that better define the intrinsic trans-family flexibility, as they account for major differences between S^GDP^ and S^GTP^ (Table 1 and Figure 3). Such an increase in flexibility of swI and swII has already been shown for Ras p21, as a feature linked to the absence of the γ-phosphate [11]. For Gα_t_, the higher flexibility of S^GDP^ resides also in the α-helical domain (Figures 3 and S1). Such a flexibility trend between inactive and active states is mostly shown also by the average Cα-Root Mean Square Deviations (Cα-RMSD; Table S1), which for all GTPases but H-Ras is higher in S^GDP^ compared to S^GTP^.

**Table 1:** Correlations between shape descriptor and PCs.

Family	State	Var^a^	R^b^	SASA_TGavg_ (Å^2^)^c^	GDP release^d^
**Arf1**	S^GDP^	91.00	0.85	91.71±20.64	0.04 [12]
	S^GTP^	65.63	0.32	8.34±2.37	
**Gα_t_**	S^GDP^	390.37	0.60	87.32±18.21	0.00072 [13]
	S^GTP^	285.11	0.40	16.85±4.48	
**Sec4**	S^GDP^	157.88	0.86	151.27±29.10	0.21 [14]
	S^GTP^	79.31	0.52	18.59±4.05	
**H-Ras**	S^GDP^	87.19	0.79	120.08±21.58	0.025 [16]
	S^GTP^	60.96	0.65	20.93±7.04	
**RhoA**	S^GDP^	111.70	0.83	107.68±20.76	0.0072 [17]
	S^GTP^	103.93	0.57	20.49±6.88	

a Total variance obtained by summing the eigenvalues from PCA.

b Correlation coefficient between SASA_TG_ and a combination of the first twenty PCs.

c SASA_TG_ index averaged over the 40000 frames constituting the 40 ns trajectories.

d Rate of GEF-independent GDP release (min^−1^); the relative bibliographic source is in square brackets.

**Figure 4:**
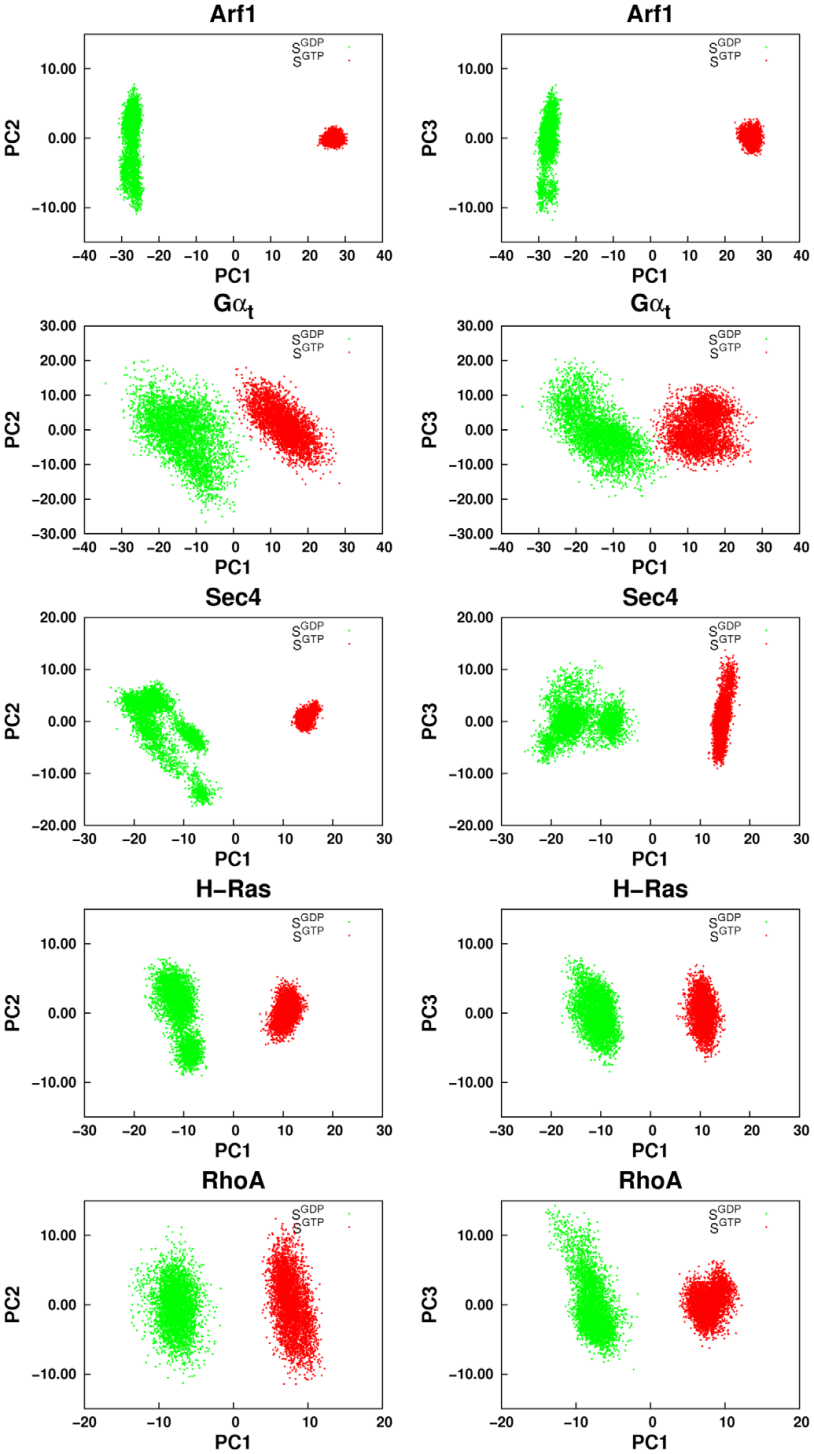
Results of PCA on the concatenated 40 ns trajectories of the inactive and active states. Frame displacements along the first three PCs derived from the concatenated trajectories of the S^GDP^ (green) and S^GTP^ (red) representatives of the Arf1, Gα_t_, Sec4, H-Ras, and RhoA families are shown. In detail, PC1 has been plotted both against PC2 (left panel) and PC3 (right panel).

The analysis of concerted motions was carried out both on single and on concatenated Cα-atom trajectories of the inactive and active forms. Consistent with the Cα-RMSF profiles, the total variance computed by summing the eigenvalues from PCA on single trajectories is always higher for the inactive forms compared to the active ones (Table 1, Figure 3). As for PCA on the concatenated trajectories, the first eigenvector (i.e. principal component 1; PC1) separated the structures visited along the trajectory into two clusters, corresponding to S^GDP^ and S^GTP^ (this separation is especially clear for all small G-proteins; Figure 4 and Figure S1). Displacements along second and third eigenvectors (PC2 and PC3) generally reflect the higher motility of S^GDP^ compared to S^GTP^ (Figure 4). For the small G proteins, Cα-atom projections along the three PCs concern collective motions of swI, swII, and inter-switch (Figures S1, S2, and S3). In contrast, for Gα_t_ the collective motions of switches and inter-switch are associated with motions of the α/β loops and of the α-helical domain (Figures S1, S2 S3).

Collectively, Ras GTPases share a similar essential dynamics that is more amplified in S^GDP^ and involves swI, swII, and inter-switch. Such a functional state-dependent dynamics is presumably linked to the nucleotide switch mechanism that pertains to S^GDP^. The additional essential motions differentiating Gα_t_ from the small G proteins may be considered as expressions of family-specific functional dynamics finalized to the nucleotide switch.

### Functional modes in the small G proteins are associated with changes in solvent accessibility of lobe 1 portions

**Figure 5:**
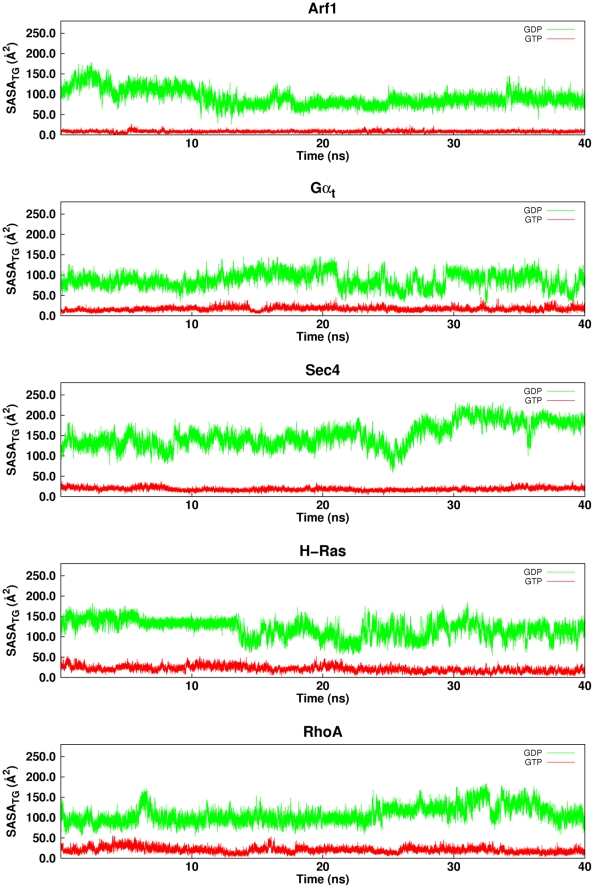
Time series of the SASA index. Time series of the SASA index computed over T^(G2∶4)^ and G^(G3∶4)^ (SASA_BP_) are shown for the S^GDP^ (green lines) and S^GTP^ (red lines) representatives of the five considered GTPases.

**Figure 6:**
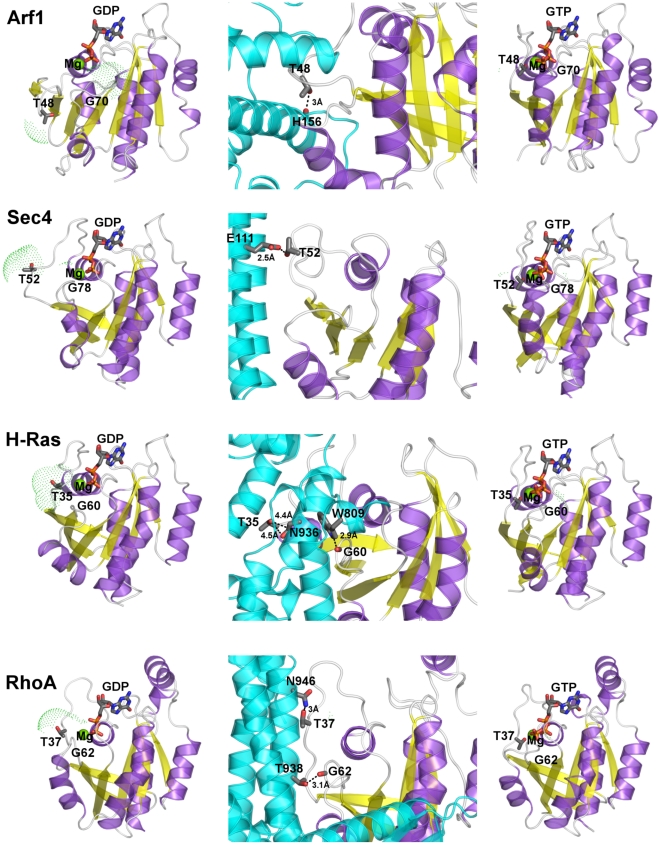
Cartoons of three different functional forms of the five GTPases. Left, central, and right panels show, respectively, the GDP-, GEF-, and GTP-bound forms of Arf1, Sec4, H-Ras, and RhoA. The GEF protein is colored cyan with helices represented as cylinders. The SASA computed on T^(G2∶4)^ and G^(G3∶4)^ is shown by green dots. The T^(G2∶4)^ side chain and the nucleotide are represented as sticks. Dashed lines indicate the distances between either the side chain oxygen atom of T^(G2∶4)^ or the backbone oxygen atom of G^(G3∶4)^ and an interacting partner on the GEF molecule.

In an attempt to find a trans-family structural indicator of the differences between S^GDP^ and S^GTP^, we computed the Solvent Accessible Surface Area (SASA) of all the highly conserved amino acids in the G-boxes (Figure 1). The analysis highlighted the SASA computed over T^(G2∶4)^ and G^(G3∶4)^ (SASA_TG_; see Figure 1B legend for the numbering explanation) as the only functional state descriptor valid for all the five GTPase families (Table 1). Indeed, the SASA_TG_ averaged over all the trajectory frames (SASA_TGavg_ in Table 1) or plotted as time series (Figure 5) is always greater for the inactive form than for the active one. This is due to the breakage of the interactions between the side chain of T^(G2∶4)^ and both the Mg^2+^ ion and a γ-phosphate oxygen atom, and between the backbone nitrogen atom of G^(G3∶4)^ and a γ-phosphate oxygen atom on going from S^GTP^ to S^GDP^ (Figure 6). Such a breakage of intermolecular interactions is expected to contribute, at least in part, to the higher flexibility of the two switches in the inactive state compared to the inactive one (Figure 3), suggesting the existence of a link between SASA_TG_ and RMSF profiles.

According to the results of Functional Mode Analysis (FMA) (see Methods), for the small G proteins SASA_TG_ correlates extremely well with the first twenty PCs derived from PCA on the single Cα-trajectories of S^GDP^, Gα_t_ showing a lower correlation coefficient (Table 1). Collectively, correlations are lower for S^GTP^ compared to S^GDP^ (Table 1).

**Figure 7:**
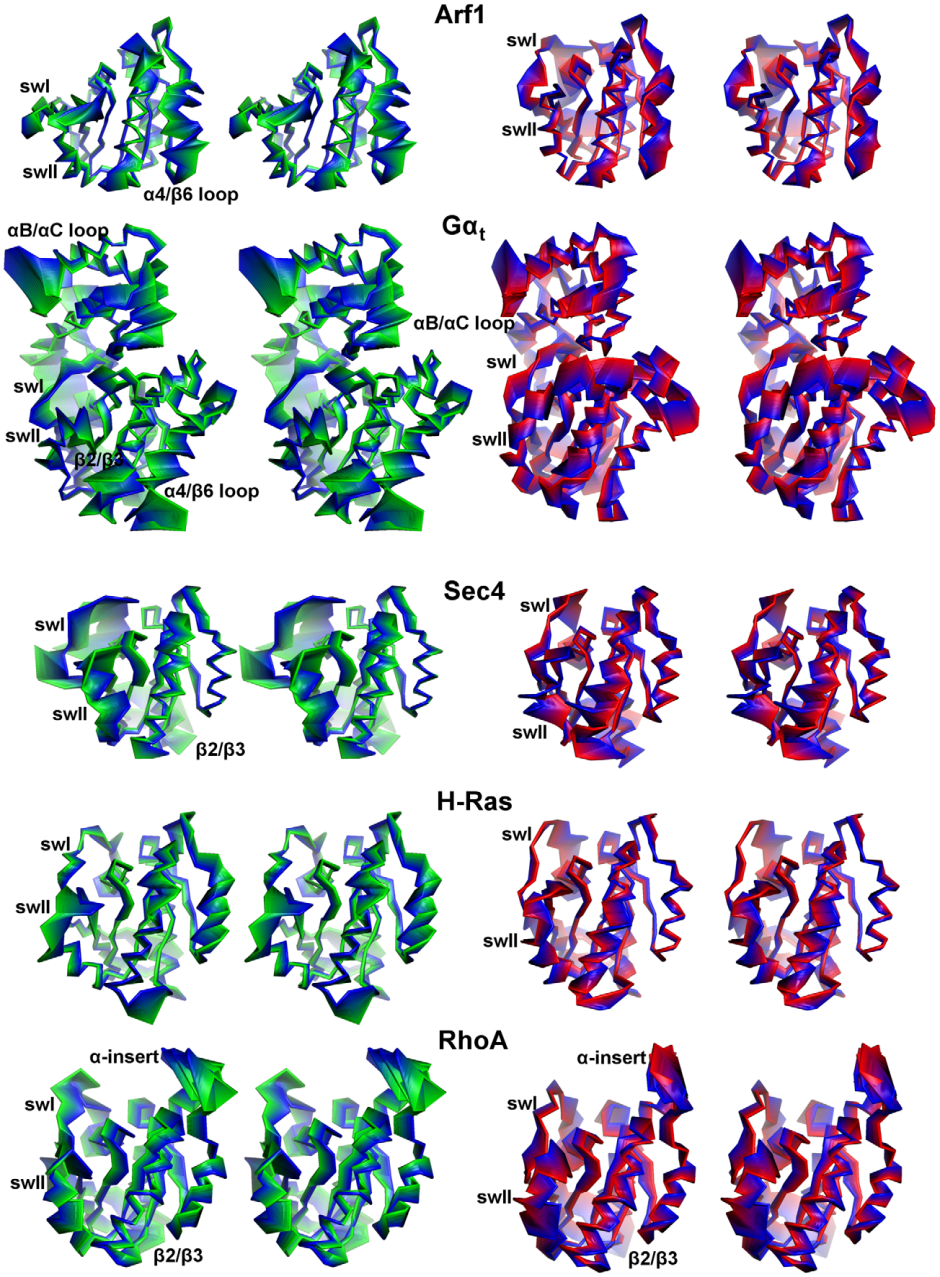
Cα-atoms projections along the first 20 PCs. The Cα-atoms projections along the linear combination of the first twenty PCs from the trajectories referred to the S^GDP^ (left) and S^GTP^ (right) states are shown.

The Cα-atom projections of the linear combination of the first twenty PCs shows that the portions that contribute the most to the combined essential motions in the S^GDP^ state include swI, swII, and inter-switch (Figure 7). Gα_t_ shows additional collective motions of α-helical domain and α4/β6 loop, whereas RhoA is characterized by additional essential motions of the α-insert (Figures 1 and 7). Remarkably, the highly conserved amino acids that contribute to SASA_TG_ lay just on swI (T^(G2∶4)^) and on swII (G^(G3∶4)^). Incidentally, in small G proteins, the movement of swI and, by a lower extent, of swII, marked, respectively, by the increase in solvent accessibility of T^(G2∶4)^ and G^(3∶4)^ on going from S^GTP^ to S^GDP^, is instrumental in GEF recognition (Figure 6 and Figure S4). The fact that those modes, which correlate with SASA_TG_, concern portions of the small G proteins implicated in GEF recognition, and that correlations pertain mostly to the S^GDP^ state, which is engineered to recognize GEFs, suggests that the correlated modes are, indeed, functional modes related to the nucleotide switch mechanism in the small G proteins. These inferences are also supported by the correlation between SASA_TGavg_ and rate of GDP release (Table 1, the linear correlation coefficient for the five considered systems being 0.86) [12]–[17]. In this respect, Sec4 shows the highest SASA_TGavg_ and GDP release rates in the absence of GEF (Table 1).

### Comparative analyses of interaction energy correlations

Further insights into the dynamic properties of S^GDP^ and S^GTP^ involved in functional specialization of selected families were gained through the analysis of correlated non-bonded interactions energies. Highly correlated interacting pairs are markers of protein regions that communicate between each other. A functional state-independent feature of the five G proteins detected by correlated energies is the lack of interaction energy correlations between β4 amino acids and any amino acid from lobe 1 (Figures S5 and S6), which reflects the low flexibility of the β-strand [6]. Other functional-state independent features, shared by Arf1 and Gα_t_, are the diffuse energy correlations involving α4/β6 loop and both lobe 1 and lobe 2 portions (Figures S5 and S6).

The patterns of correlated interaction energies show that the representative members of the five G protein families considered in this study, less evident in Arf1, share intra-lobe 1 energy correlations in their inactive state, which are lost upon nucleotide exchange. On the contrary, inter-lobe energy coupling between α3, on one side, and swII and inter-switch, on the other one, is more marked in S^GTP^ compared to S^GDP^. A singularity of H-Ras with respect to the other small G proteins is the energy coupling between β2/β3 turn and C-term of α5, which is more marked in S^GDP^ compared to S^GTP^ (Figure S5).

Family specialization in the context of the energetic coupling between amino acid pairs is more evident by comparing Gα_t_ with the four small G proteins (Figures S5 and S6). In this respect, a singularity of Gα_t_ is the extended coupling between C-term of α5 and lobe 1 portions in S^GDP^, which is lost upon activation (Figure S6). Other singularities of the inactive state of Gα_t_ is the inter-domain coupling between αF/linker1, on one side, and inter-switch, swII, α3, α4/β6 loop and α5, on the other, which is more marked in the inactive state. Finally, in S^GDP^ of Gα_t_, the α/β loops that are involved in GEF recognition show more marked energy coupling compared to the β/α loops. Taken together, these features reflect the bi-domain structural organization of the protein as well as the singular GEF recognition mode and GEF-catalyzed nucleotide switch mechanism, which makes the difference from the small G proteins.

We also analyzed functional-state dependent changes in the patterns of the amino acid pairs whose interaction energies correlate with the pairwise interaction energies involving the nucleotide (i.e. amino acid pairs energetically coupled with nucleotide-mediated interaction(s)). Incidentally, the average interaction energy profile of the nucleotide in the two different functional states of the five representatives shows remarkable trans-family conservation (Figure S7). In more detail, for both the S^GDP^ and S^GTP^ forms of the small G proteins, the G1 and G4 boxes (Figure 1) give, respectively, the first and second strongest contributions in terms of attractive interaction energy values, whereas the G2 and G3 boxes contribute only in the S^GTP^ state. Peculiar to both functional forms of Gα_t_ is the fact that the G2 box gives the second strongest contribution due to R174^(G2∶1)^.

**Figure 8:**
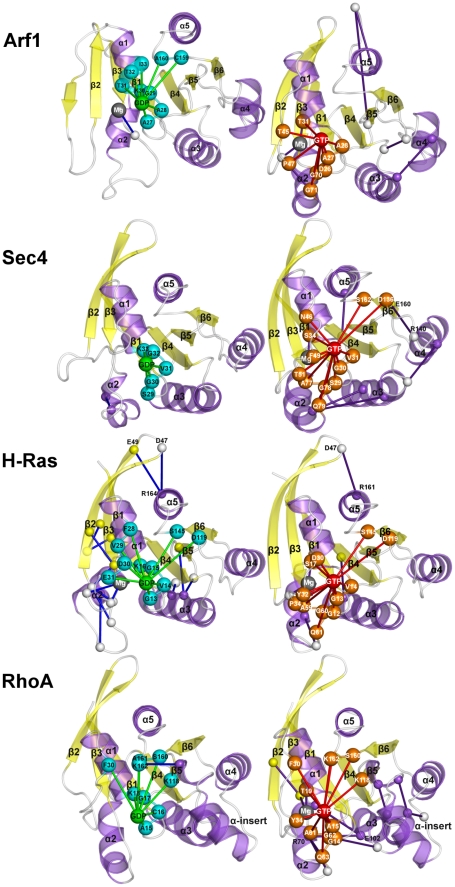
Nucleotide-protein interaction energies correlated with protein-protein interaction energies. Cartoons of the S^GDP^ (left panels) and S^GTP^ (right panels) states of the four small G proteins are shown. Proteins are colored according to secondary structure. Correlated amino acid pairs are indicated by spheres centered on the Cα-atoms and connected by lines. GDP and GTP are, respectively, represented as green and red spheres centered on the ribose C4′ atom. The spheres concerning the amino acids of the GDP and GTP binding sites are cyan and orange, respectively, whereas that concerning the Mg^2+^ ion is gray. Lines that involve the nucleotide sphere are green and red for the S^GDP^ and S^GTP^ forms, respectively. The spheres concerning the correlated amino acid pairs not directly involved in interaction with the nucleotide are white, smaller than those of the nucleotide binding site, and connected by blue and violet lines in the S^GDP^ and S^GTP^ states, respectively. For Arf1, coupled amino acids pairs are found between α3 and α4, between α3/β5 loop and α5 (C-term), and within the C-term of the S^GTP^ state. For Sec4, the almost absent correlated pairs in the S^GDP^ form are replaced by interactions between swII and α3, between α3 and α4, between α3/β5 loop and α5 C-term, and between β5/α4 loop and β6 (Figure 8). Remarkably, the latter amino acid pair, energetically coupled with the pair S29^(G1∶3)^-GTP, involves R140 in the β5/α4 loop and E160^(G5∶1)^ conserved in the Sec4, H-Ras, and RhoA sequences. Similar to the other small G proteins, RhoA activation, tends to increase the swII-α3 correlated connectivities, which include the R70^(swII∶7)^-E102^(α3∶14)^ ion pair. The latter presumably contributes to increase the α3-bending already observed in the S^GDP^ state. Other coupled pairs in the active form locate on the α-insert.

In general, in S^GDP^ of the small G proteins, the amino acids involved in direct interaction with the nucleotide essentially lay on α1 and the P-loop, whereas in S^GTP^, the nucleotide interaction sphere extends to swI and swII amino acids (Figure 8). Upon activation, for Arf1, Sec4, and RhoA the energy coupling between nucleotide binding site and other protein portions increases. In contrast, for H-Ras, GTP substitution for GDP reduces the network of electrostatic interactions energetically coupled with GDP found between the β2/β3 hairpin and α5 (i.e. including the salt bridges between D47 in the β2/β3 turn and both R161^(α5∶10)^ and R164^(α5∶13)^ and between E49^(β3∶-2)^ and R164^(α5∶13)^; Figure 8).

**Figure 9:**
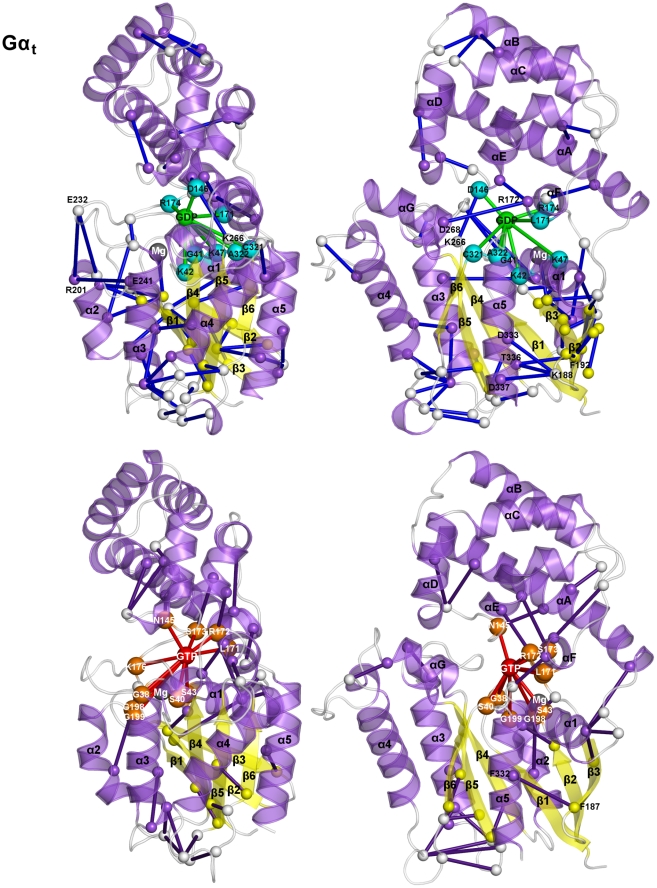
Nucleotide-protein interaction energies correlated with protein-protein interaction energies for Gα_t_. The explanation of this Figure is the same as that of Figure 8, with the difference that in this Figure two different side views for each functional form of Gα_t_ are shown. Intra-Ras correlated pairs are both intra-lobe and inter-lobe located. In deep detail, intra-lobe 1 pairs which are close to the nucleotide binding site, are located: a) between β2 and β3 strands; b) between swI and swII; c) intra-swII; and d) between swII and β3. Different from the intra-lobe 1 pairs, intra-lobe 2 pairs are essentially distal from the nucleotide. Some of them locate between α3, on one side, and β4/α3, α3/β5 loop, α4/β6 loop as well as α4, on the other one. Other intra-lobe 2 pairs involve β5 and α4/β6 loop as well as α4 and β6/α5 loop. Inter-lobe correlated pairs essentially involve swII, R201^(swII∶1)^ being paired with both E241^(α3∶5)^ (corresponding to E102^(α3∶5)^ in Arf1) and E232 in the β4/α3 loop. Other noticeable inter-lobe correlated pairs, distal from the nucleotide binding site, involve the β2/β3 hairpin and α5. In detail, K188 in the β2/β3 turn is involved in correlated pairs with D333^(α5∶6)^, T336^(α5∶9)^, and D337^(α5∶10)^, whereas F192^(β3∶3)^ is paired with T336^(α5∶9)^.

The energetic coupling in Gα_t_ shows a singular behavior compared to the small G proteins (Figures 8 and 9). In fact, both S^GDP^ and S^GTP^ show a significantly higher number of correlated pairs compared to the small G proteins (Figure 9). These pairs are both inter- and intra-domain located and undergo a change in distribution upon activation (Figure 9). As for S^GDP^, inter-domain correlated pairs essentially involve R172^(αF∶6)^ and the fully conserved D268^(G4∶4)^, which interacts also with the nucleotide, as well as D146 (in the αD/αE loop) and K266 (in the β4/α3 loop) (Figure 9). As for the intra-α-helical domain-located pairs, only ten amino acid pairs correlate with the nucleotide interaction(s). Finally, intra-Ras correlated pairs are both intra-lobe and inter-lobe located (see Figure 9 legend for deep detail). Activation causes a general reduction in energy coupling, which is more significant in lobe 1 and between the two lobes of the Ras-like domain. Indeed, the correlated pairs characterizing S^GTP^ are essentially located in lobe 2 portions distal from the nucleotide (i.e. in α3, α4 as well as the α3/β5 and α4/β6 loops (Figure 9)).

Collectively, the analysis of correlated interaction energies highlights the presence of an allosteric mechanism associated with the nucleotide switch in Gα_t_ but not in the small G proteins.

### Comparative analysis of dynamic domains

**Figure 10:**
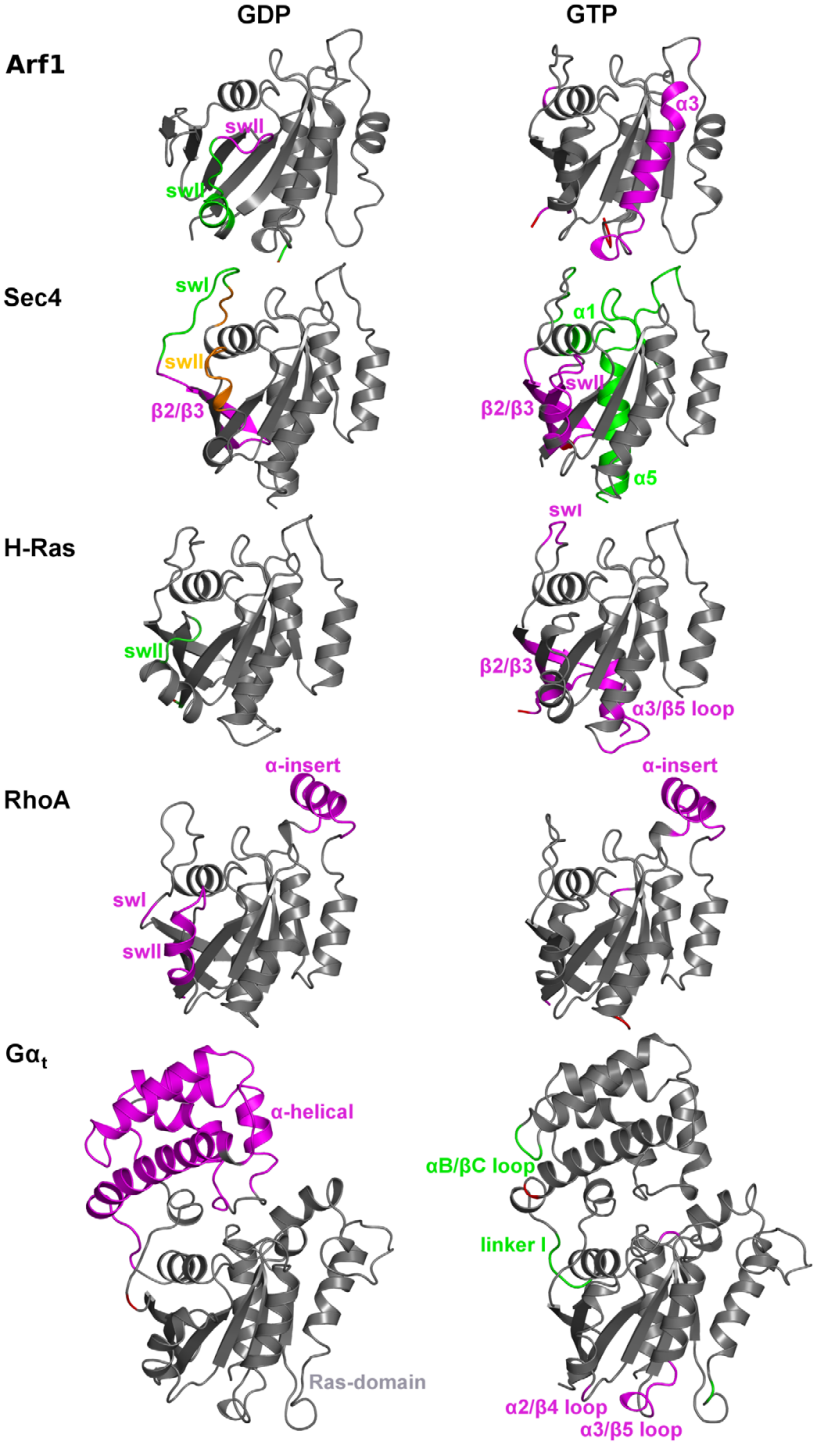
Domain representation according to Dynamic Domain analysis. Cartoon representations of the S^GDP^ (right panel) and S^GTP^ (left panel) representatives of the five families are shown. The coloring scheme highlights protein portions that belong to mechanically coherent domains. The first (i.e. the biggest one) domain is gray, the second is magenta, the third is green, the fourth is orange and the fifth is red. Portions clustered separately from the first domain are labeled accordingly.

The five G proteins (in both active and inactive forms) were also compared by the Dynamic Domain (DD) method, which clusterizes the Cα-atoms of the system according to their propensity to be part of mechanically coherent domains in the trajectory frames [18]. Not surprisingly, the first cluster separates active vs inactive G-proteins, stressing the existence of differential effects of GDP and GTP binding on G protein dynamics. In S^GDP^ of the small G proteins, mechanically distinct domains include swI and swII, which are directly implicated in GEF recognition and the nucleotide switch, whereas S^GTP^ does not show common clustering patterns. Furthermore, the S^GDP^ of Gα_t_ shows unique features since the α-helical domain appears mechanically distinct from the Ras-like domain (Figure 10).

## Discussion

MD simulations done in this study helped unraveling the functional dynamics of Ras superfamily GTPases, providing atomistic details that were not reached by previous evolutionary or coarse-grained studies [6]. Indeed, current simulations clearly show that the intrinsic flexibility of S^GDP^ is higher than that of S^GTP^, suggesting that GEF recognition and nucleotide switch mechanism require higher amplitude motions than effector recognition or GTP hydrolysis.

Novel trans-family features pertaining to functional dynamics were inferred from the analysis of interaction energy correlations. The latter revealed intra-lobe 1 correlations in all the five G proteins; such correlations attenuated upon activation. This may relate with lobe 1 being heavily involved in function-retention dynamics. Furthermore, interaction energy correlations highlighted almost complete lack of correlations involving the β4 segment in both functional states of the five G proteins. This feature remarks the bi-lobate nature of the conserved Ras-like domain, which is related to β4 being the holder of the strongest and most conserved hinge point [6].

The results of this study suggest that, in small G proteins, functional modes, i.e. collective motions directly related to function, are more evident in the inactive state rather than in the active one. These modes, which involve swI and swII in lobe 1, correlate with changes in solvent exposure of T^(G2∶4)^ and G^(G3∶4)^, which, in the small G proteins, with emphasis on T^(G2∶4)^, are involved in GEF recognition. These results, together with the existence of a correlation between SASA_TGavg_ and rate of GDP release, suggest that the two conserved G-box amino acids participate either directly or indirectly in the mechanism of nucleotide switch in small G proteins. Functional modes involving swI and swII are less detectable for Gα proteins, suggesting that biologically relevant modes in large and small G proteins are different. Indeed, the analyses of essential motions and dynamic domains support previous inferences that in Gα proteins the nucleotide switch involves concerted motions of the α-helical domain with respect to the Ras-like domain, following allosteric GEF recognition by the α/β loops [19]. Consistent with these results, the inactive state of Gα_t_ is characterized by a significantly more extensive communication between nucleotide binding site and distal regions involved in GEF recognition compared to the small G proteins. This was inferred from S^GDP^ of Gα_t_ showing an evident energy coupling between nucleotide binding site and distal portions like the β2/β3 turn, α5, and the three α/β loops, which participate in GEF recognition [3]. Remarkably, this energy coupling is absent in S^GTP^, in which function, i.e. effector binding and GTP-hydrolysis, does not require long distance communication as it involves regions like swI and swII that participate in the nucleotide binding site. Thus, the energy coupling between nucleotide and GEF binding sites likely pertains to the nucleotide switch mechanism in Gα proteins and not in small G proteins. The evident communication between nucleotide binding site and inter-switch-C term of Gα_t_ is in line with the results of previous simulations of the receptor-G protein complex suggesting that the receptor-induced detachment of α5 from the inter-switch promotes a cascade of structural changes in Gα that propagate from the C-term to the α-helical domain through lobe 1 portions of the Ras-like domain [19]. These changes, indeed, culminate with the formation of a nucleotide exit route in between the αF-helix and β6/α5 loop [19].

The analysis of the interaction energy correlations highlights novel family-specific features. These include interaction energy correlations involving α4/β6 loop and the remaining portions of Arf1 and Gα_t_, related to the higher length and flexibility of this loop in the two proteins compared to the other three G proteins. Moreover, a singularity of H-Ras compared to the other small G proteins is a reduction in energy coupling between nucleotide binding site and distal regions like α5, on going from S^GDP^ to S^GTP^. This may relate, at least in part, to the postulated implication of inter-switch and α5 of H-Ras in a novel switch mechanism involving a nucleotide-dependent change in the membrane anchoring of the protein, operated through α4 and the hypervariable region (HVR) in α5 [20].

In summary, in Ras GTPases, the intrinsic dynamics oriented to functional specialization essentially pertains to the inactive state rather than to the active one and clearly separates the small G proteins from Gα_t_. Indeed, mechanically distinct domains implied in the mechanism of nucleotide switch could be detected in the presence of GDP but not in the presence of GTP. S^GDP^ is engineered to respond to the GEF's request to release GDP and this follows different mechanisms in the small G proteins compared to the members of the Gα family. Whereas in small G proteins GEF binds directly to swI and swII, which are implicated in nucleotide exit, in the Gα family GEF binds to protein portions that are distal from the nucleotide, thus implying allosteric communication for GEF-catalyzed nucleotide release. The latter is expected to involve also a concerted motion of the α-helical domain with respect to the Ras-like domain, feature intrinsic to S^GDP^ and amplified by GEF. In contrast, for S^GTP^ of all G proteins, in which nucleotide and effector binding sites are quite close, the functioning mechanism does not require long distance communication nor inter-domain motions that are, hence, attenuated compared to S^GDP^. In this respect, the intrinsic flexibility of small G proteins and Gα proteins shares more commonalties in the active state compared to the inactive one.

Current and previous results [6] suggest that the Ras superfamily utilizes a hierarchical organization of its structural flexibilities; lobe 1 motions associated with its switching function must be retained in order to accomplish the primary G protein function of changing its affinity for GEFs, effector proteins, and GAPs through different bound nucleotides, but additional motions across both lobes of the protein are family-specific and play a role in determining the unique functional characteristics of specific members. Molecular communication between lobe 1 and lobe 2 portions (intra-Ras communication) and between α-helical and Ras-like domains, for Gα_t_, represents the way to accomplish functional specialization.

Taken as a whole the results of this investigation reveal that the topology of the conserved Ras-like domain is such that it allows for differential flow of information depending on the bound nucleotide, i.e. GDP enhances the flexibility of mechanically distinct portions involved in nucleotide switch, and favors long distance allosteric communication (in Gα proteins), compared to GTP.

## Methods

### Simulated proteins

The following S^GDP^ and S^GTP^ representatives of the Arf1, Gα_t_, Sec4, H-Ras, and RhoA families were selected as input of MD simulations: Arf1 (PDB codes: 1HUR and 1O3Y), Gα_t_ (PDB codes: 1TAG and 1TND), Sec4 (PDB codes: 1G16 and 1G17), H-Ras (PDB codes: 4Q21 and 5Q21), and RhoA (PDB codes: 1FTN and 1KMQ) based on the following criteria: i) homogeneity of the molecular specie, i.e. same sequence, for the two functional states, and ii) existence of a crystal structure for the isolated state, i.e. not in complex with other proteins. In a few cases, wild-type structures (in one or both S^GDP^ and S^GTP^ states) were generated by manipulation of mutant structures: L71Q mutant for Arf1 S^GTP^, N25F, and N25F/L63Q for the S^GDP^, and S^GTP^ states of RhoA, respectively. Due to the intrinsic GTPase activity, the GTP-bound forms had been crystallized in complex with an hydrolysis-resistant analogue of GTP, i.e. GppNHp for 5P21, 1G17, and 1KMQ, and GTPγS for 1TND. In our simulations the GTP analogues were converted to GTP.

Finally, all the simulated systems contained the Mg^2+^ ion together with the coordinating water molecules. In the case of the GDP-bound form of Sec4, the Mg^2+^ ion substitutes for the Co^2+^ ion originally present in the 1G16 structure. The two different metal ions are expected to be interchangeable with no substantial structural effects [21].

### Set-up of MD simulations

MD simulations on the five representative Ras GTPases (Arf1, Gα_t_, Sec4, H-Ras, and RhoA) were carried out using the GROMACS4 simulation package [22] with the AMBER03 all atoms force field [23], [24], by using the TIP3P water model to describe the solvent. AMBER parameters to describe the GDP and GTP molecules were taken from literature [25]. Depending on the dimensions of the systems, a variable number of Na^+^ and Cl^−^ ions placed at optimum electrostatic positions were added in order to neutralize the system (Table S1). Periodic Boundary Conditions (PBC) were applied using an octahedric box as a unit cell, imposing a minimum distance of 12 Å between the solute and the box boundaries.

All the input crystallographic structures were subjected to energy minimization keeping restricted the positions of main chain atoms, all the Cβ atoms, the nucleotide, the Mg^2+^ cation and the coordinating water molecules. The systems were then equilibrated at 300 K for 4 ns of backbone-restricted MD simulations. The Particle Mesh Ewald (PME) method was employed to compute the electrostatic interactions. Short range repulsive and attractive interactions were computed using a Lennard-Jones potential with a cutoff of 10 Å. The LINCS algorithm [26] was used to constrain all bond lengths except those in water molecules, allowing for an integration time step of 2 fs by the leap-frog algorithm. The v-rescale thermostat [27] was employed to keep the system at a constant temperature of 300 K, by using a coupling constant (τ_t_) of 0.1 ps. The pressure of the system was kept fixed at 1 atm, using the Berendsen weak coupling algorithm [28] with a coupling constant (τ_p_) of 1 ps. The pre-equilibrated systems were then released for 1 ns prior to 40 ns of unrestrained isothermal-isobaric (T = 300K, P = 1 atm) MD simulations.

For each system, trajectory replicas of 40 ns were achieved by reassigning velocities to the frame extracted from the 20000^th^ frame of the original trajectory. Due to overall consistency between original and replicated trajectories, this work shows only the results of the original one.

### Analysis of trajectories

Trajectories were subjected to a variety of analyses. RMSDs, RMSF, and SASA were performed using the Wordom software [29]. Detailed information concerning the essential motions of the proteins was obtained by using Essential Dynamics (ED) analyses. In this respect, covariance matrices (Cα-atoms in our case) were built, using either isolated or concatenated MD trajectories, and diagonalized to a set of eigenvectors (describing the essential deformation modes) and associated eigenvalues (which explain the amount of each-mode variance). As for ED on the concatenated trajectories, the Cα-atom structure averaged over all the concatenated frames was used as a reference for the building of the co-variance matrix. In order to facilitate comparison along different proteins, a minimum consensus length for the inactive and active states was chosen for each family, i.e. 18-177 for Arf1, 29-339 for Gα_t_, 20-183 for Sec4, 3-165 for H-Ras, and 5-178 for RhoA.

The search for correlations between structural features and essential modes was carried out through the FMA [30] tool, by using the Linear Mutual Information (LMI) estimator [31]. In this framework, a number of size/shape and intermolecular interaction descriptors were correlated with linear combinations of a variable number of PCs.

The dynamical/mechanical properties of the simulated systems were characterized by means of the DD method implemented into the PCASUITE package (http://mmb.pcb.ub.es/software/pcasuite/) [32].

Non bonded interaction energies involving each protein residue, the nucleotide, and the Mg^2+^ ion were monitored every 20 ps along the trajectory. Only the non bonded interactions whose average values along the simulations were greater than 2 kcal mol^−1^ were considered relevant for the analysis and were processed to find potential pair-correlations according to the following equation [33]:
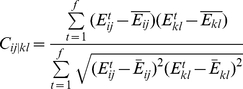
(1)Where 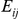 and 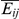 (as well as 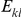 and 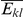) stand for instantaneous (i.e. frame^th^-associated) and average interaction energy values between particles i and j (as well as k and l). A *N* × *N* correlation matrix is thus built, where *N* is the total number of relevant interacting pairs. Only the elements with absolute correlation coefficient ≥0.4 were further considered in the analysis. Note that by mapping the inter-atomic energy correlation matrix into the residue space, a residue correlation matrix can be derived:
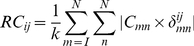
(2)where N is the dimension of the energy correlation matrix and *k* is the number of relevant interactions, whose correlation value is above the correlation threshold, in which residues *i* and *j* are involved. 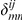 is 1 if residue *i* and *j* are involved in interactions with *m* and *n* (*n* and *m*), otherwise 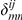 is set to 0.

## Supporting Information

Figure S1 Cα-atoms projections along PC1. Cα-atoms projections along the PC1 from the trajectories obtained by concatenating the trajectories relative to the S^GDP^ (left panel) and S^GTP^ (right panel) bound representatives of the five families are shown. A number of conformations were generated by displacing the Cα-atoms of the first frame of the S^GDP^ and S^GTP^ trajectories from the minimum to the maximum displacements observed along PC1 in the relative cluster. For S^GDP^ and S^GTP^, the color changes, respectively, from green to blue, and from red to blue. Labels mark the structural portions in the Ras-like domain, which are more involved in such displacements.
(3.13 MB TIF)

Figure S2 Cα-atoms projections along PC2. Cα-atoms projections along the PC2 from the trajectories obtained by concatenating the trajectories relative to the S^GDP^ (left panel) and S^GTP^ (right panel) bound representatives of the five families are shown. See the legend to Figure S1 for an explanation of this figure.
(3.22 MB TIF)

Figure S3 Cα-atoms projections along PC3. Cα-atoms projections along the PC3 from the trajectories obtained by concatenating the trajectories relative to the S^GDP^ (left panel) and S^GTP^ (right panel) bound representatives of the five families are shown. See the legend to Figure S1 for an explanation of this figure.
(3.11 MB TIF)

Figure S4 Superimposed structures of the S^GDP^ (green), S^GTP^ (red), and GEF-bound forms of the four small G proteins.
(2.64 MB TIF)

Figure S5 Interaction energy correlations for the small G proteins. The inter-residue interaction energy correlation matrices for Arf1, Sec4, H-Ras, and RhoA are shown. The dimension of each symmetric matrix corresponds to the number of residues shared by the two functionally different states of the protein. Each column or row represents a specific residue. The regions above and below the matrix main diagonal concern S^GDP^ and S^GTP^, respectively. The secondary structure elements are shown, following nomenclature and color code described in Figure 1. Interaction energy and correlation coefficient cutoffs of 2 kcal mol^-1^ (in absolute value) and ≥0.4, respectively, were employed in the analysis. The color scale is from 0 (blue) to 1 (red), where 0 corresponds to a 0.4 correlation coefficient.
(0.81 MB TIF)

Figure S6 Interaction energy correlations for Gα_t_. The inter-residue interaction energy correlation matrix for Gα_t_ is shown. The dimension of the symmetric matrix corresponds to the number of residues shared by the two functionally different states of the protein. Each column or row represents a specific residue. The regions above and below the matrix main diagonal concern S^GDP^ and S^GTP^, respectively. The secondary structure elements are shown, following nomenclature and color code described in Figure 1. Interaction energy and correlation coefficient cutoffs of 2 kcal mol^-1^ (in absolute value) and ≥0.4, respectively, were employed in the analysis. The color scale is from 0 (blue) to 1 (red), where 0 corresponds to a 0.4 correlation coefficient.
(0.71 MB TIF)

Figure S7 Average interaction energy profiles of the nucleotide. Green and red lines refer to S^GDP^ and S^GTP^, respectively of Arf1, Gα_t_, Sec4, H-Ras, and RhoA. The secondary structure elements are shown on the abscissa, following nomenclature and color code described in Figure 1.
(0.55 MB TIF)

Table S1 Details of the simulated systems and average Cα-RMSD.
(0.04 MB PDF)
