# Supplementary figures and images for: Nucleotide Binding Switches the Information Flow in Ras GTPases
Source: PLoS Comput Biol. 2011 Mar 3;7(3):e1001098. doi: 10.1371/journal.pcbi.1001098 (PMC3048383; doi:10.1371/journal.pcbi.1001098)

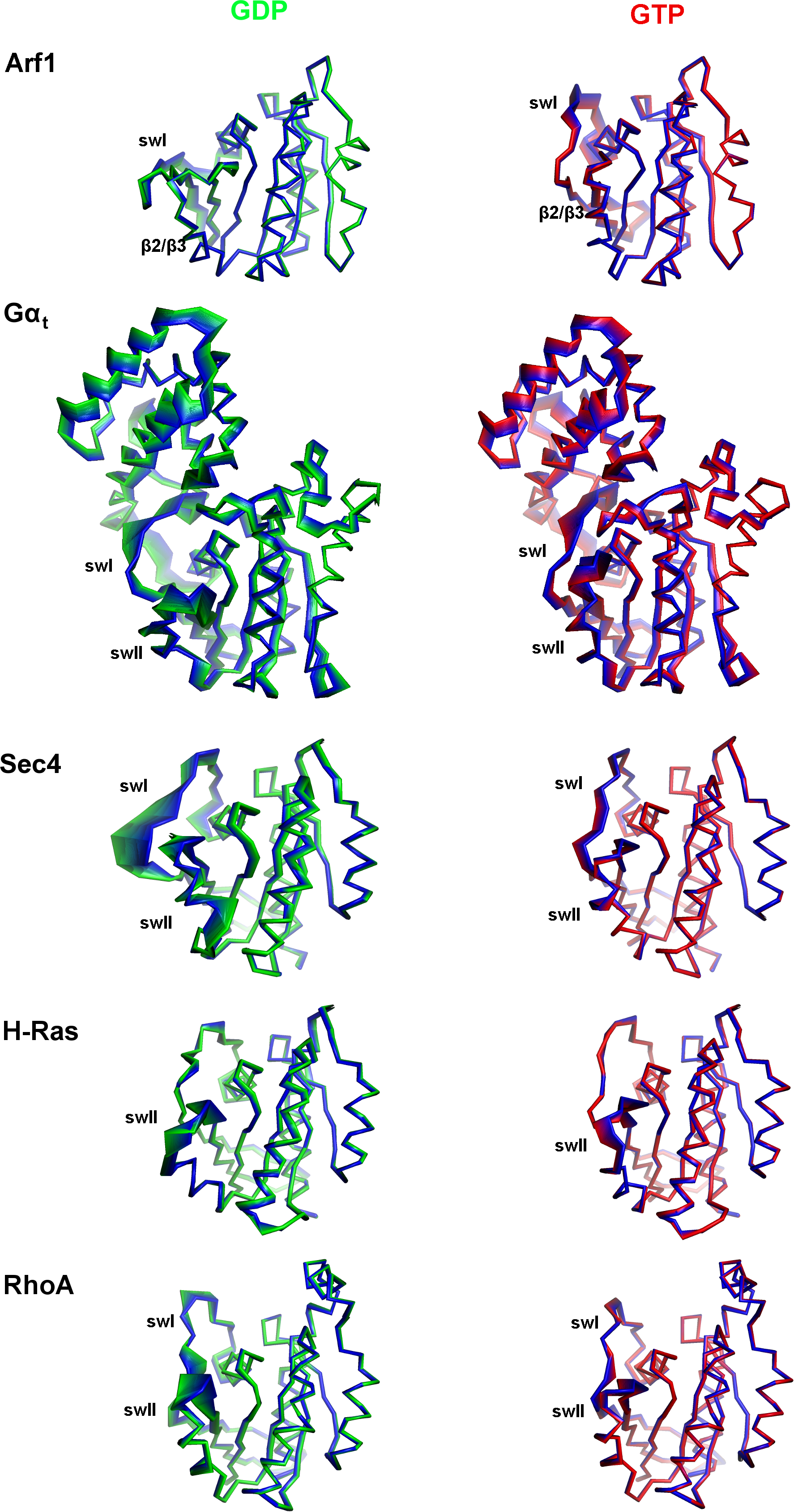

Supplement: Figure S1 — Cα-atoms projections along PC1. Cα-atoms projections along the PC1 from the trajectories obtained by concatenating the trajectories relative to the SGDP (left panel) and SGTP (right panel) bound representatives of the five families are shown. A number of conformations were generated by displacing the Cα-atoms of the first frame of the SGDP and SGTP trajectories from the minimum to the maximum displacements observed along PC1 in the relative cluster. For SGDP and SGTP, the color changes, respectively, from green to blue, and from red to blue. Labels mark the structural portions in the Ras-like domain, which are more involved in such displacements. (3.13 MB TIF) [file pcbi.1001098.s001.tif]

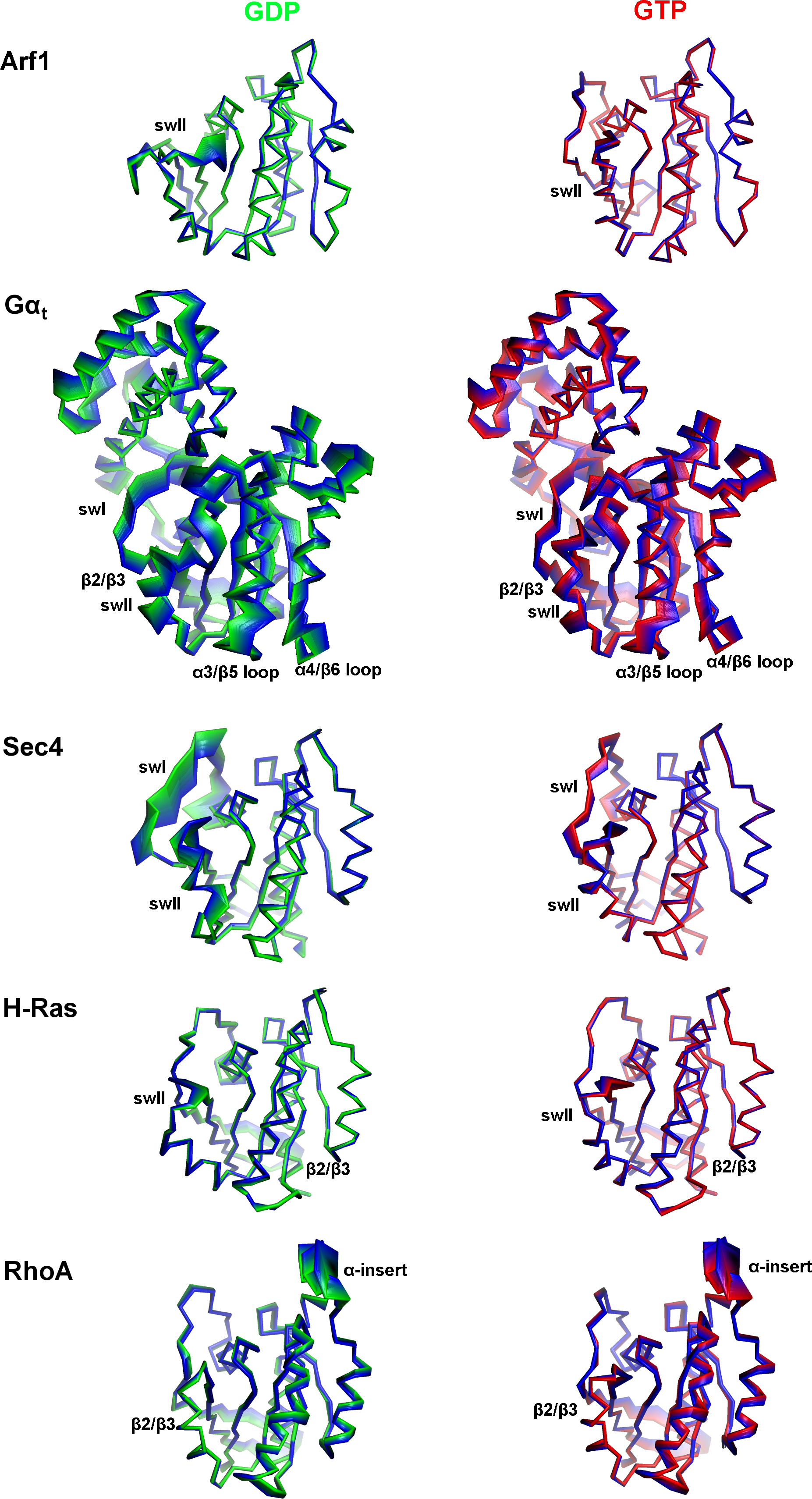

Supplement: Figure S2 — Cα-atoms projections along PC2. Cα-atoms projections along the PC2 from the trajectories obtained by concatenating the trajectories relative to the SGDP (left panel) and SGTP (right panel) bound representatives of the five families are shown. See the legend to Figure S1 for an explanation of this figure. (3.22 MB TIF) [file pcbi.1001098.s002.tif]

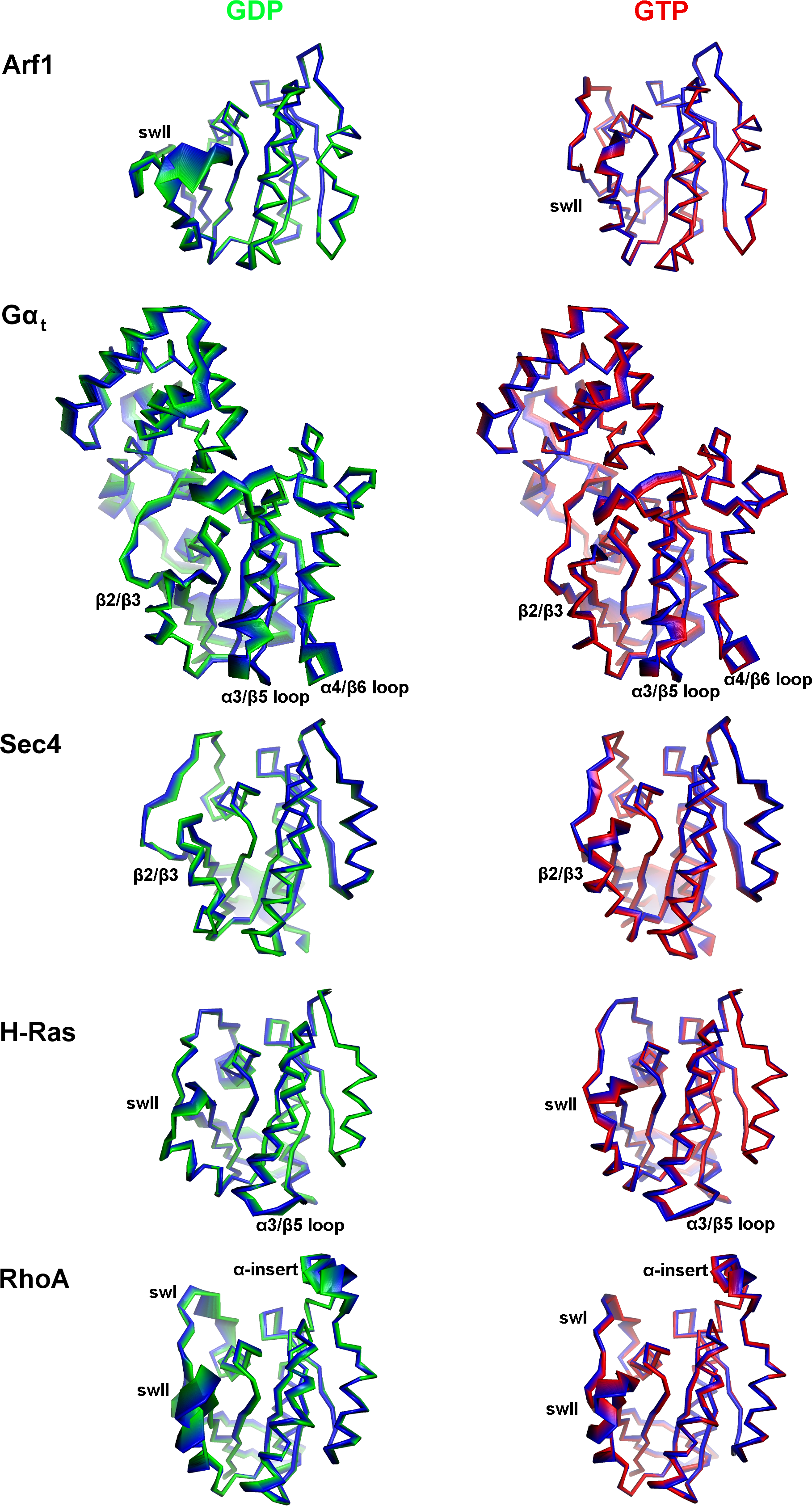

Supplement: Figure S3 — Cα-atoms projections along PC3. Cα-atoms projections along the PC3 from the trajectories obtained by concatenating the trajectories relative to the SGDP (left panel) and SGTP (right panel) bound representatives of the five families are shown. See the legend to Figure S1 for an explanation of this figure. (3.11 MB TIF) [file pcbi.1001098.s003.tif]

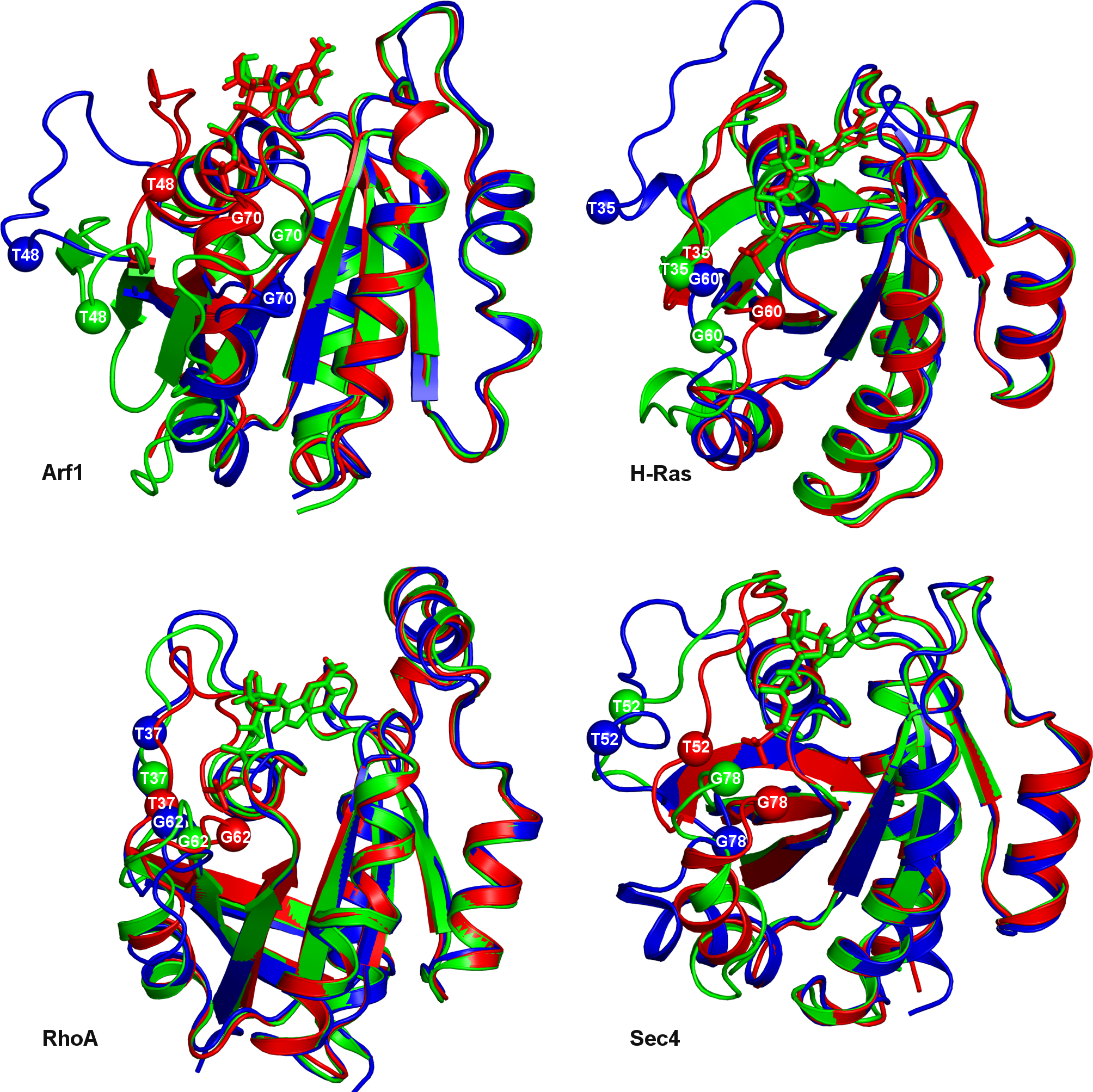

Supplement: Figure S4 — Superimposed structures of the SGDP (green), SGTP (red), and GEF-bound forms of the four small G proteins. (2.64 MB TIF) [file pcbi.1001098.s004.tif]

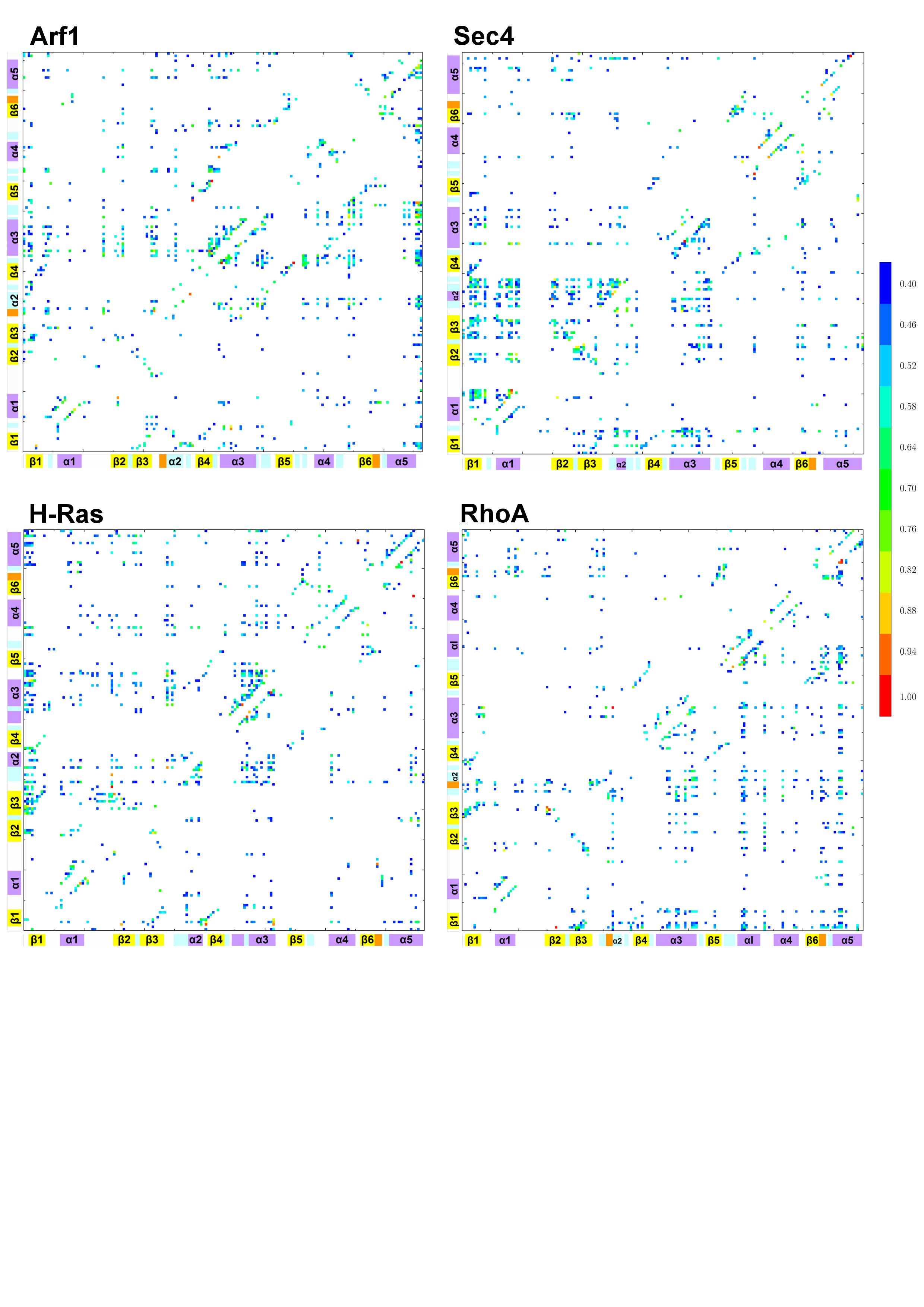

Supplement: Figure S5 — Interaction energy correlations for the small G proteins. The inter-residue interaction energy correlation matrices for Arf1, Sec4, H-Ras, and RhoA are shown. The dimension of each symmetric matrix corresponds to the number of residues shared by the two functionally different states of the protein. Each column or row represents a specific residue. The regions above and below the matrix main diagonal concern SGDP and SGTP, respectively. The secondary structure elements are shown, following nomenclature and color code described in Figure 1. Interaction energy and correlation coefficient cutoffs of 2 kcal mol-1 (in absolute value) and ≥0.4, respectively, were employed in the analysis. The color scale is from 0 (blue) to 1 (red), where 0 corresponds to a 0.4 correlation coefficient. (0.81 MB TIF) [file pcbi.1001098.s005.tif]

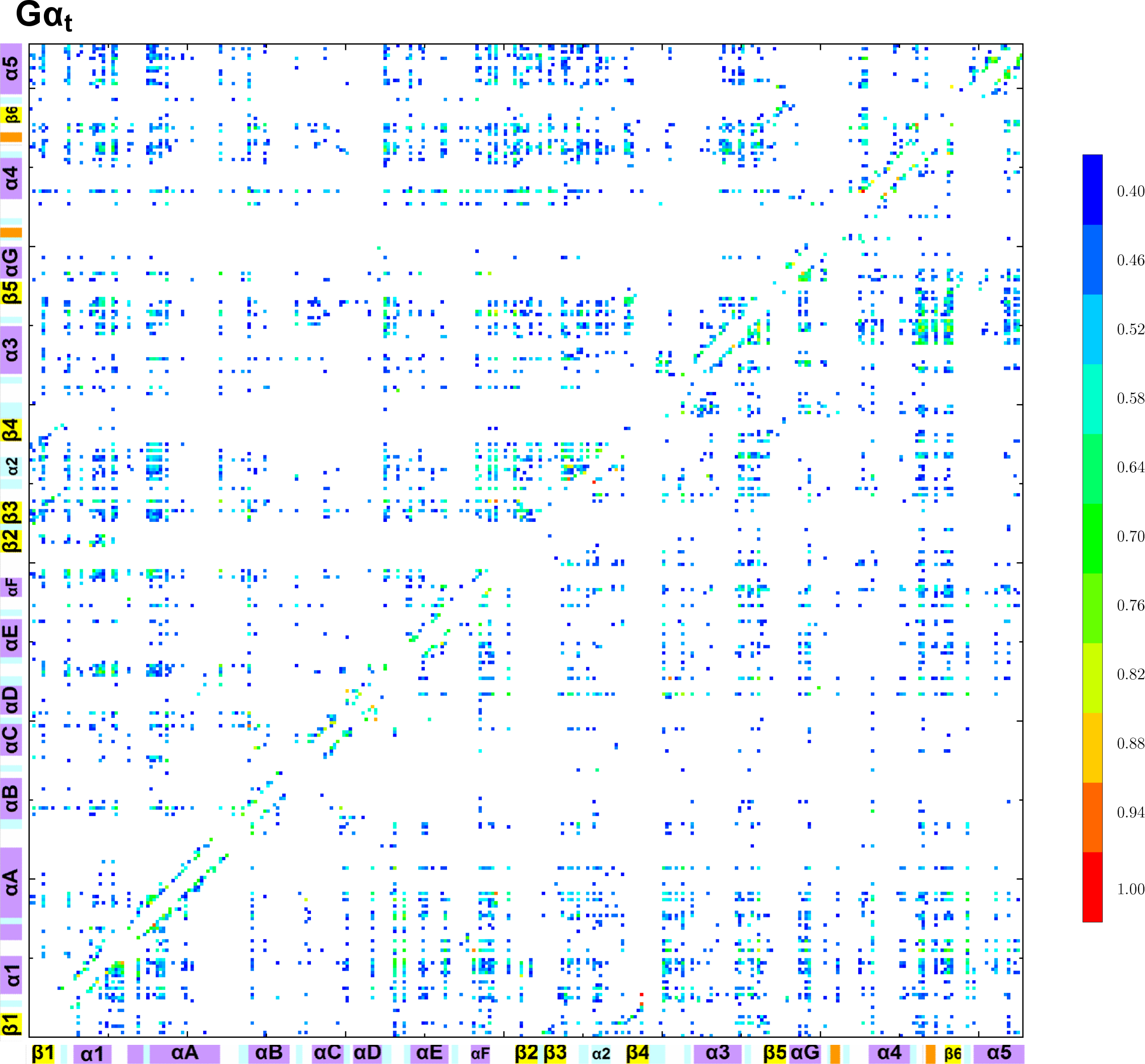

Supplement: Figure S6 — Interaction energy correlations for Gαt. The inter-residue interaction energy correlation matrix for Gαt is shown. The dimension of the symmetric matrix corresponds to the number of residues shared by the two functionally different states of the protein. Each column or row represents a specific residue. The regions above and below the matrix main diagonal concern SGDP and SGTP, respectively. The secondary structure elements are shown, following nomenclature and color code described in Figure 1. Interaction energy and correlation coefficient cutoffs of 2 kcal mol-1 (in absolute value) and ≥0.4, respectively, were employed in the analysis. The color scale is from 0 (blue) to 1 (red), where 0 corresponds to a 0.4 correlation coefficient. (0.71 MB TIF) [file pcbi.1001098.s006.tif]

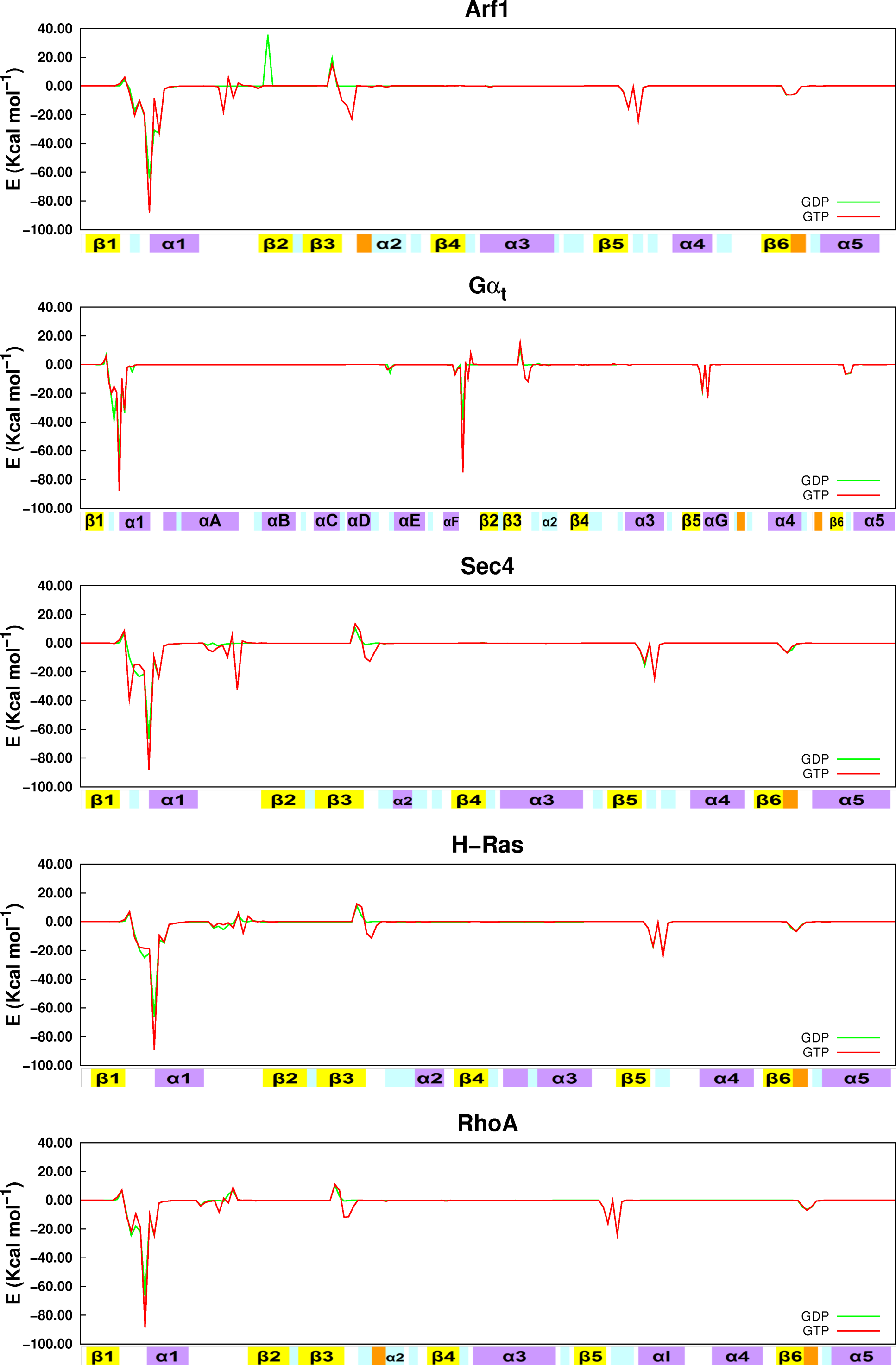

Supplement: Figure S7 — Average interaction energy profiles of the nucleotide. Green and red lines refer to SGDP and SGTP, respectively of Arf1, Gαt, Sec4, H-Ras, and RhoA. The secondary structure elements are shown on the abscissa, following nomenclature and color code described in Figure 1. (0.55 MB TIF) [file pcbi.1001098.s007.tif]
