# Supplementary material for: Nucleotide Binding Switches the Information Flow in Ras GTPases
Source: PLoS Comput Biol. 2011 Mar 3;7(3):e1001098. doi: 10.1371/journal.pcbi.1001098 (PMC3048383; doi:10.1371/journal.pcbi.1001098)
Supplement: Table S1 — Details of the simulated systems and average Cα-RMSD. (0.04 MB PDF) [file pcbi.1001098.s008.pdf]

Table S1. Details of the simulated systems and average Cα-RMSD.

c	Chain[b]	aa[c]	Box size (Å)[d]	H_2O[e]	Na^+[f]	Cl^-[g]	Tot. atoms[h]	$RMSD_{avg}$[i]
**1HUR**	A	2-181	72.8x72.8x72.8	8883	21	18	29635	1.251±0.101
**1O3Y**	A	16-181	77.1x77.1x77.1	10917	27	21	35511	0.961±0.082
**1TAG**	A	27-340	96.1x96.1x96.1	21003	50	41	68177	1.759±0.246
**1TND**	C	27-342	96x96x96	20862	51	41	67796	1.371±0.181
**4Q21**	A	1-168	71.3x71.3x71.3	8370	24	17	27857	0.994±0.100
**5P21**	A	1-166	70x70x70	7808	25	16	26134	1.038±0.087
**1G16**	C	19-185	71.13x71.13x71.13	8269	21	17	27573	1.287±0.307
**1G17**	A	19-186	75.5x75.5x75.5	8916	23	18	29532	0.867±0.155
**1FTN**	A	4-180	82x82x82	13158	34	26	42396	1.239±0.172
**1KMQ**	A	4-180	81.2x81.2x81.2	12692	34	25	41001	1.132±0.142

[a]PDB code of the input structure
[b]PDB chain selected as an input.
[c]Sequence length of the input structure.
[d]Dimensions of the box.
[e]Number of solvating water molecules.
[f]Number of sodium ions.
[g]Number of chlorine ions.
[h]Total number of atoms in the box.
[i] Cα-RMSD averaged over the 40000 trajectory frames.
